# Comprehensive Analysis of *GH3* Gene Family in Potato and Functional Characterization of *StGH3.3* under Drought Stress

**DOI:** 10.3390/ijms242015122

**Published:** 2023-10-12

**Authors:** Panfeng Yao, Chunli Zhang, Tianyuan Qin, Yuhui Liu, Zhen Liu, Xiaofei Xie, Jiangping Bai, Chao Sun, Zhenzhen Bi

**Affiliations:** 1 State Key Laboratory of Aridland Crop Science, Gansu Agricultural University, Lanzhou 730070, China; yaopf@gsau.edu.cn (P.Y.); zhangchunl@st.gsau.edu.cn (C.Z.); qinty@st.gsau.edu.cn (T.Q.); lyhui@gsau.edu.cn (Y.L.); liuzhen@gsau.edu.cn (Z.L.); xiexf@st.gsau.edu.cn (X.X.); baijp@gsau.edu.cn (J.B.); sunc@gsau.edu.cn (C.S.); 2 College of Agronomy, Gansu Agricultural University, Lanzhou 730070, China

**Keywords:** potato, GH3 family, water deficit, expression pattern

## Abstract

As an important hormone response gene, Gretchen Hagen 3 (GH3) maintains hormonal homeostasis by conjugating excess auxin with amino acids during plant stress-related signaling pathways. *GH3* genes have been characterized in many plant species, but they are rarely reported in potato. Here, 19 *StGH3* genes were isolated and characterized. Phylogenetic analysis indicated that StGH3s were divided into two categories (group I and group III). Analyses of gene structure and motif composition showed that the members of a specific StGH3 subfamily are relatively conserved. Collinearity analysis of *StGH3* genes in potato and other plants laid a foundation for further exploring the evolutionary characteristics of the *StGH3* genes. Promoter analysis showed that most *StGH3* promoters contained hormone and abiotic stress response elements. Multiple transcriptome studies indicated that some *StGH3* genes were responsive to ABA, water deficits, and salt treatments. Moreover, qRT-PCR analysis indicated that *StGH3* genes could be induced by phytohormones (ABA, SA, and MeJA) and abiotic stresses (water deficit, high salt, and low temperature), although with different patterns. Furthermore, transgenic tobacco with transient overexpression of the *StGH3.3* gene showed positive regulation in response to water deficits by increasing proline accumulation and reducing the leaf water loss rate. These results suggested that *StGH3* genes may be involved in the response to abiotic stress through hormonal signal pathways. Overall, this study provides useful insights into the evolution and function of *StGH3s* and lays a foundation for further study on the molecular mechanisms of *StGH3s* in the regulation of potato drought resistance.

## 1. Introduction

As the first plant hormone discovered more than 70 years ago, auxin plays an important role in the control of cell elongation and division, tropic responses to light, general root and shoot architecture, organ patterning, and responses to biotic and abiotic stimuli [1,2]. Auxin homeostasis and the auxin response pathway are regulated by several groups of auxin-responsive genes. Among them, auxin/indoleacetic acid (Aux/IAA), Gretchen Hagen 3 (GH3), and small auxin up RNA (SAUR) are considered to be early auxin-responsive gene families [3]. The GH3 protein is involved in hormone homeostasis in vivo through the conjugation of amino acids to the free forms of salicylic acid (SA), jasmonic acid (JA), and indole-3-acetic acid (IAA). The *GH3* gene was first discovered in soybean (*Glycine max*) in 1984 [4], and the functional study of the plant *GH3* gene family has become a hot topic.

In plants, the *GH3* gene is a key component of the hormone mechanism that regulates growth and development in response to both biological and abiotic stresses. The *GH3* family genes are involved in the formation of amide synthase, which maintains the dynamic balance of hormones by catalyzing the binding of amino acids with IAA, SA, and JA [5]. The first *GH3* gene was isolated from soybean by differential hybridization screening as an auxin-induced cDNA clone from etiolated hypocotyls [4]. Subsequently, a large number of *GH3* homologs have been found in many plants, such as Arabidopsis [5], rice [6], wheat [7], maize [8], soybean [9], cucumber [10], tomato [11], apple [12], etc. Phylogenetic studies have shown that the *GH3* gene family has been classified into three groups (I–III) based on sequence similarity and substrate specificities [5]. Members of group I can adenylate jasmonic acid (JA) in vitro and have JA–amino synthetase activity, whereas group II members are able to adenylate IAA and catalyze IAA conjugation to amino acids through amide bonds [13]. Therefore, groups I and II are involved in the homeostasis of JA and IAA, respectively, through the conjugation of the hormones to amino acids. However, no adenylation activity on the substrates tested was found for group III members, and group III *GH3* genes are absent in rice [14]. One common feature of these protein is that they can accept different amino acids as substrates. In the case of auxin conjugates, the characteristics of amino acids used in the reaction determine whether the generated molecules are degraded, stored or become signal compounds [15]. The function of protein is related to the properties of its substrate, but several physiological processes are usually affected by protein activity, such as light and stress response [16].

More and more evidence has shown that most *GH3* gene promoters contain auxin-responsive cis-acting elements (AuxRE) and other stress-related elements [17], indicating that they may participate in plant growth and development and stress responses through hormonal pathways. Therefore, the biological functions of several members of the *GH3* gene family have been studied in detail [18]. Some *GH3* genes were reported to be involved in controlling plant growth and development. *WES1*, an Arabidopsis *GH3* gene, mediates phytochrome B-regulated light signals in hypocotyl growth [19]. An *AtGH3.9* mutation promotes primary root growth and influences the crosstalk between the auxin and jasmonate signal pathways [20]. *ydk1-D*, an auxin-responsive *GH3* mutant, is involved in hypocotyl and root elongation through the regulation of auxin activity [21]. Overexpression of *AtGH3.5* induced by ABA, SA, and auxin results in an auxin-resistant phenotype for transgenic plants [22]. A GH3-like gene, *CcGH3*, was isolated from pepper fruit, which is regulated by auxin and ethylene and plays a role in the ethylene pathway involved in fruit ripening [23]. Besides their roles in growth and development, *GH3* family genes also play crucial roles in plant resistance to stresses. *OsGH3.2*, a GH3 family member in rice, affects cold and drought tolerance by regulating ABA content [24]. Activation of *OsGH3.13* reduces the auxin content to enhance the drought tolerance of rice and alters its root architecture [25]. In cotton, silencing the *GH3.5* gene reduces the drought and salt tolerance of transgenic cotton. *AtGH3.5* is a potential activator in the immune response of Arabidopsis, participating in SA mediated defense reactions through NPR1 dependent and independent pathways [26]. Arabidopsis GH3-like defense gene 1 is required for the accumulation of SA, activation of defense responses, and resistance to *Pseudomonas syringae* [27]. Overall, all these studies suggest that GH3-mediated auxin homeostasis is an important constituent of auxin signaling in response to various stresses. 

Potato (*Solanum tuberosum* L.) is very important to global food security and the fourth largest staple food in the world. This crop is mainly planted in the northwest of China, and the long-term water shortage in the northwest makes China potatoes often suffer from drought stress, which seriously affects the local and even national potato commodity output [28]. In recent years, research on drought resistance genes and functional identification of potato has become a hot spot in recent years. For instance, the overexpression of *StGA2ox1* [29], *StDRO1* [30], *StPIP1* [31], *StRFP2* [32], *StMAPK11* [33], and *StProDH1* [34] genes has effectively improved the adaptability of transgenic plants to drought stress. However, the comprehensive information and expression patterns of *GH3* family genes responding to abiotic stresses in potato are largely unknown, and the mechanism of *GH3* gene has not yet been reported. In this study, the whole genome of *GH3* gene family members in potato was identified, and the conserved motif, gene structure, phylogenetic relationship, chromosome location and cis-acting elements of *StGH3* gene were analyzed. In addition, the gene replication events and collinearity with other species were also analyzed. Finally, the *StGH3.3* gene which significantly responded to drought stress was identified, and its drought resistance function was verified by tobacco transient transformation. This study laid a solid foundation for the functional analysis of stgh3 gene, and it is also a candidate gene for genetic improvement of potato drought resistance.

## 2. Results

### 2.1. Identification of StGH3 Genes

In this study, 19 *StGH3* genes were identified in potato by two BLAST methods based on the known GH3 domain sequences, and the redundant genes were removed. To better distinguish these genes, the *StGH3* genes were randomly named *StGH3.1*–*StGH3.19* (Table S1). Subsequently, the physical and chemical properties of the *StGH3* genes were predicted, such as length of coding sequence (CDS), isoelectric point (pI), molecular weight (MW), and subcellular localization. The lengths of the proteins range from 303 (StGH3.19) to 614 (StGH3.13) amino acids (aa), with an average of 530 aa (Table S2). Their MWs range from 33.94 KDa (StGH3.19) to 69.53 KDa (StGH3.13), with an average of 59.99 KDa. The lowest *p*I is 4.78 (StGH3.14), and the highest *p*I is 8.76 (StGH3.19), with an average of 5.94. The predicted subcellular localization results indicated that 12 StGH3 proteins are located in the cytoplasm, 6 StGH3 proteins are located in the nucleus, and 1 StGH3 protein is located in the mitochondria. 

### 2.2. Phylogenetic Analysis Divided StGH3s into Two Sub-Groups

**Figure 1:**
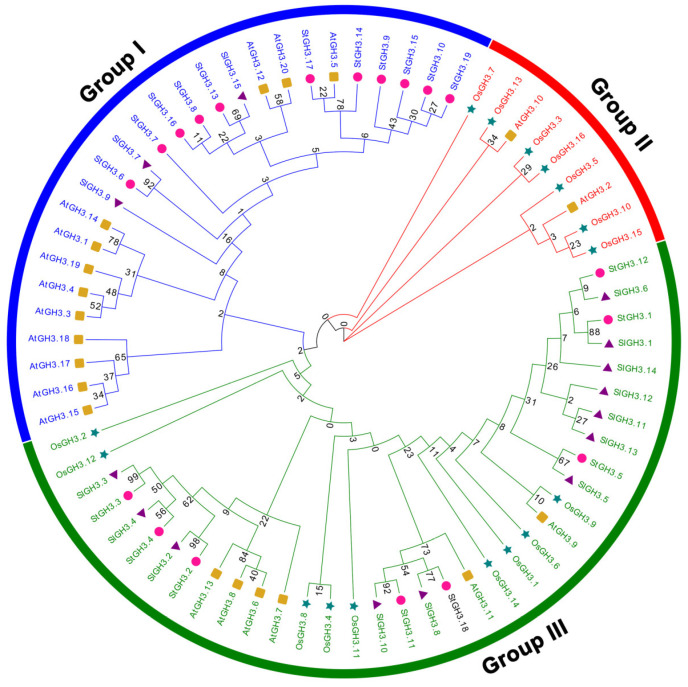
Unrooted phylogenetic tree for 19 StGH3s, 20 AtGH3s, 15 SlGH3s, and 16 OsGH3s was constructed by MEGA7.0. The neighbor connection (NJ) method was used to construct the phylogenetic tree, and the default parameter value was set to 1000.

A phylogenetic tree was constructed using the maximum likelihood (ML) method to better understand the evolutionary relationship between the GH3 family of potato and other species, including 19 StGH3s, 20 AtGH3s, 15 SlGH3s, and 16 OsGH3s. The tree showed that all GH3 proteins were divided into three major groups (I, II, and III), which is consistent with previous publications (Figure 1). A total of 11 StGH3s (3.6, 3.7, 3.8, 3.9, 3.10, 3.13, 3.14, 3.15, 3.16, 3.17, and 3.19) were classified into group I, and 8 StGH3s (3.1, 3.2, 3.3, 3.4, 3.5, 3.18, 3.11, and 3.12) were classified into group III. Interestingly, there were no StGH3 members clustered into group II. We found that StGH3s were more concentrated in group I, while the StGH3s of group III were relatively uniformly distributed in each sub-branch. In addition, StGH3s were more closely related to tomato than Arabidopsis and rice. In the smallest subfamily, StGH3s and SlGH3s formed 10 sister pairs (StGH3.13–SlGH3.15, StGH3.6–SlGH3.7, StGH3.12–SlGH3.6, StGH3.1–SlGH3.1, StGH3.5–SlGH3.5, StGH3.18–SlGH3.8, StGH3.11–SlGH3.10, StGH3.2–SlGH3.2, StGH3.4–SlGH3.4, and StGH3.3–SlGH3.3), but there was only 1 pair (StGH3.17–SlGH3.5) between potato and Arabidopsis, and no StGH3s and OsGH3s clustered together. These results indicated that the functions of *StGH3* genes in potato are diverse, which is consistent with the reports in Arabidopsis and rice.

### 2.3. Structural Analysis and Motif Composition of StGH3 Genes

**Figure 2:**
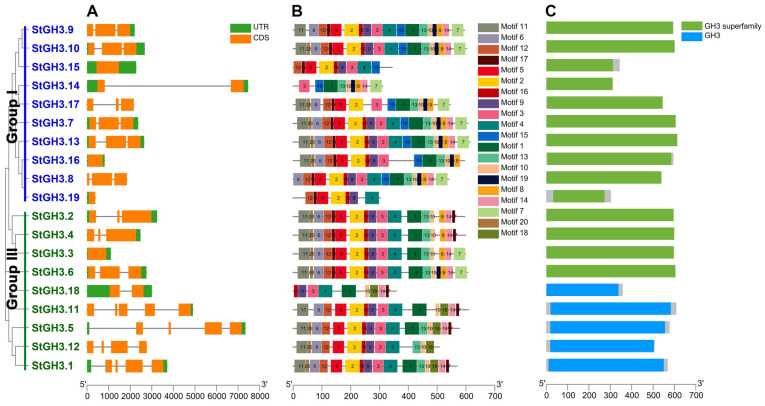
The phylogenetic relationship, conserved motifs, and gene structure of StGH3. (**A**) A phylogenetic tree and map of the exon–intron structure were constructed. The green box represents the untranslated 5′ and 3′ regions; the orange box represents exons; the black line represents introns. (**B**) The conserved motifs of StGH3 proteins. (**C**) The conserved domain of StGH3 proteins.

To further understand the structural composition of *StGH3* genes, the exon/intron structures of *StGH3s* were determined by comparing the genomic DNA sequences (Figure 2A). The results showed that most of the coding sequences of *StGH3* genes (16/19) are disrupted by introns, and the number of introns ranges from 1 to 4, demonstrating that there are some differences among the 19 *StGH3* genes. Among them, only *StGH3.14* and *StGH3.18* contain one intron, and the rest of the *StGH3* genes contain at least two introns. The genes with the largest number of introns are *StGH3.1*, *StGH3.5*, and *StGH3.11*, which belong to the same subfamily. Interestingly, there are fewer differences in exon/intron structure between different subfamilies of *StGH3*, which is slightly different from previous studies. In general, members of the same subfamily have similar gene structures.

To further explore the characteristic regions and predict the function of *StGH3*, 20 conserved motifs were identified through online MEME and further visualized by TBtools (Figure 2B and Figure S1). The length of these motifs varies between 11 (motifs 17 and 20) and 50 (motifs 1) aa. The number of motifs in StGH3 proteins varies from 7 to 19, of which 14 are functionally defined. Motifs 1 to 14 together constitute the GH3 conserved sites [7]. In fact, the motifs of StGH3 are extremely conserved, and motifs 11, 6, 12, 17, 5, 2, 16, 9, 3, and 4 exist in almost all StGH3s. Except for StGH3.15, StGH3.14, StGH3.19, and StGH3.18, all the other proteins contain at least 14 motifs. Of course, there were also some differences between the two groups. For example, motif 15 exists only in group I, while motif 18 is unique to group III, suggesting that these two motifs may play important roles in the function of different subfamilies. Finally, the analysis of the conserved domains showed that StGH3 protein sequences all contain extremely conserved GH3 domains or GH3 superfamily domains (Figure 2C). Overall, the conserved motif composition and similar gene structures within the same groups of *GH3* members, coupled with the results of the phylogenetic analysis, supported the reliability of the population classification.

### 2.4. Chromosomal Distribution, Gene Duplication, and Synteny Analysis of StGH3 Genes

A chromosomal distribution map of *StGH3* genes was generated using the latest potato genome database (Figure S2). A total of 19 *StGH3* genes were unevenly distributed in 6 of the 12 potato chromosomes. Among them, Chr10 contains the most *StGH3* genes (10, ~53%), followed by Chr7 (3, ~16%), Chr1, and Chr2, which each contain two genes (~11%), and Chr5 and Chr10 containing only one (~5%). Interestingly, the distribution of *StCH3* genes on the chromosomes is mostly concentrated at the two ends of the chromosomes, and very few *StGH3* genes are distributed in the middle of the chromosome.

**Figure 3:**
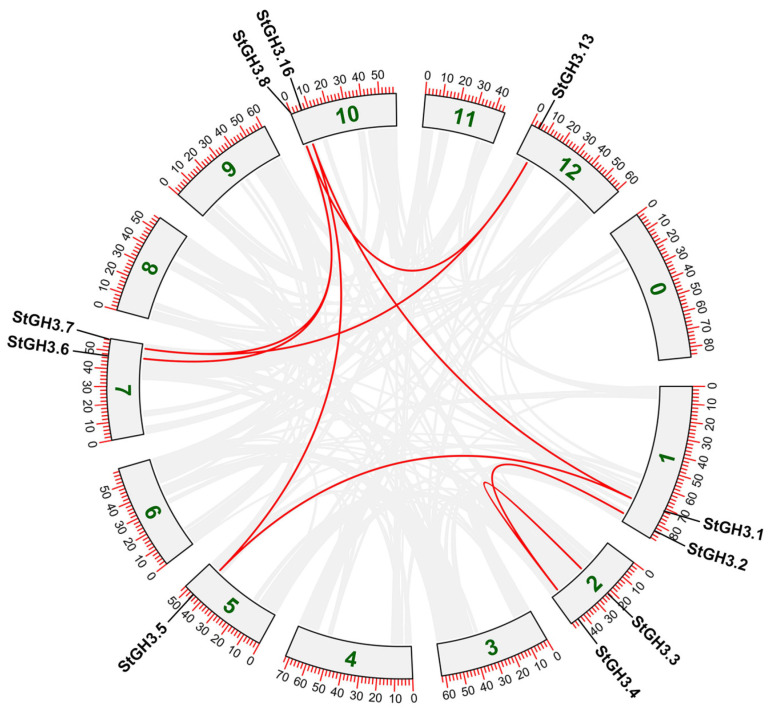
The relationship between *StGH3* gene-containing chromosomes was visualized through multiple collinear scanning toolkits (MCScanX) and TBtools. Gray lines represent collinear blocks within potato genome, while the red lines highlight *GH3* gene pairs. 0-12 represents the chromosomes of potato.

Since gene duplication plays an important role in the occurrence of new functions and the expansion of gene families, we further analyzed the duplication events involving the *StGH3* genes, including tandem and segmental duplication events. Duplication of a chromosomal region in which two or more genes are within 200 kb is defined as a tandem duplication event [35]. The results showed that four *StGH3* genes are clustered into two tandem repeat event regions (*StGH16*/*StGH17*, *StGH15*/*StGH19*) in Chr10, which indicated that they are the hot spots for *StGH3* gene distribution. Meanwhile, nine pairs of segmental duplication genes were detected between six chromosomes: Chr1 (*StGH3.1*)/Chr10 (*StGH3.12*), Chr1 (*StGH3.2*)/Chr2 (*StGH3.4*), Chr1 (*StGH3.1*)/Chr5 (*StGH3.5*), Chr10 (*StGH3.8*)/Chr12 (*StGH3.13*), Chr10 (*StGH3.12*)/Chr5 (*StGH3.5*), Chr10 (*StGH3.16*)/Chr7 (*StGH3.6*), Chr10 (*StGH3.8*)/Chr7 (*StGH3.7*), Chr12 (*StGH3.13*)/Chr7 (*StGH3.7*), and Chr2 (*StGH3.3*)/Chr2 (*StGH3.4*) (Figure 3). In summary, these results suggested that certain *StGH3* genes may have been produced by gene duplication and that tandem duplication events may have been the main driving force in the evolution of *StGH3.*

### 2.5. Evolutionary Analysis of StGH3 Genes among Multiple Species

**Figure 4:**
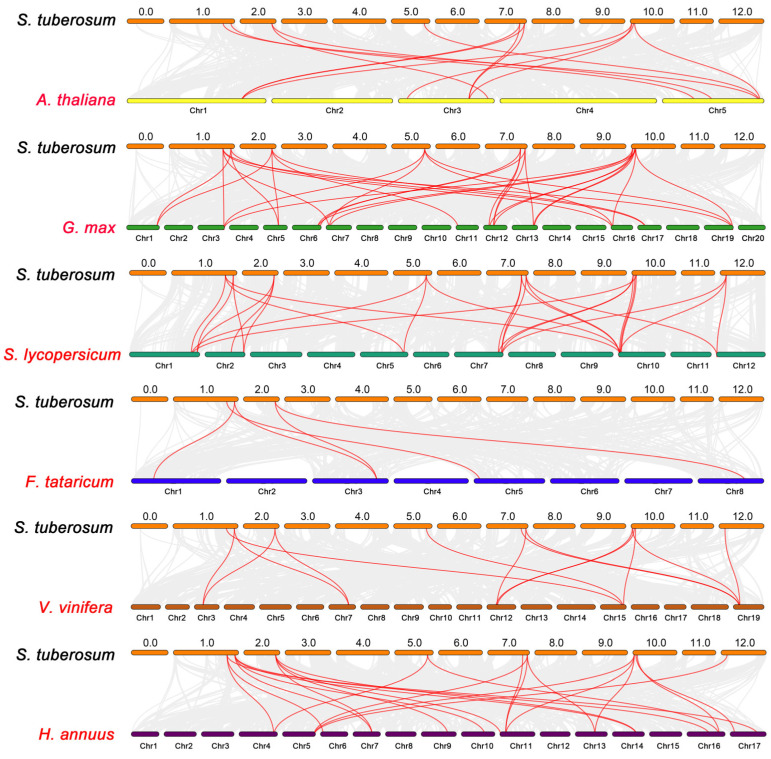
Collinearity analysis of *GH3* genes between potato and six other plant species. Gray lines represent collinear blocks in potato genome and other plant genomes, and red curves represent collinear *GH3* genes.

To further explore the evolutionary relationship of *StGH3* genes, a phylogenetic tree consisting of six dicotyledonous species (Arabidopsis, tomato, soybean, grape, sunflower, and tartary buckwheat) was constructed (Figure 4). A total of 10 *StGH3* genes are homologous to tomato, grape, and sunflower, followed by Arabidopsis and soybean with 9, and tartary buckwheat with only 3. The numbers of homologous pairs of the other six species (Arabidopsis, soybean, tomato, tartary buckwheat, grape, and sunflower) are 13, 30, 26, 5, 14, and 22, respectively. *StGH3* genes have the most gene pairs with soybean: *StGH3.1*, *StGH3.4*, *StGH3.5*, and *StGH3.12* have four syntenic gene pairs with soybean; *StGH3.2*, *StGH3.6*, *StGH3.7*, and *StGH3.8* have three pairs; and *StGH3.11* has two pairs. Furthermore, we found that *StGH3.4* has the most syntenic gene pairs (17) among all the genes, followed by *StGH3.2* (14), *StGH3.1* (13), *StGH3.8* (13), *StGH3.7* (12), *StGH3.5* (11), *StGH3.6* (10), *StGH3.12* (9), *StGH3.11* (6), and *StGH3.13* (5), suggesting that *StGH3.4* may play a key role in the evolution of the *GH3* family. In addition, the number of gene pairs between potato and other species is not related to the genome size of the other species. 

### 2.6. Analysis of Cis-Acting Elements of StGH3 Promoters

**Figure 5:**
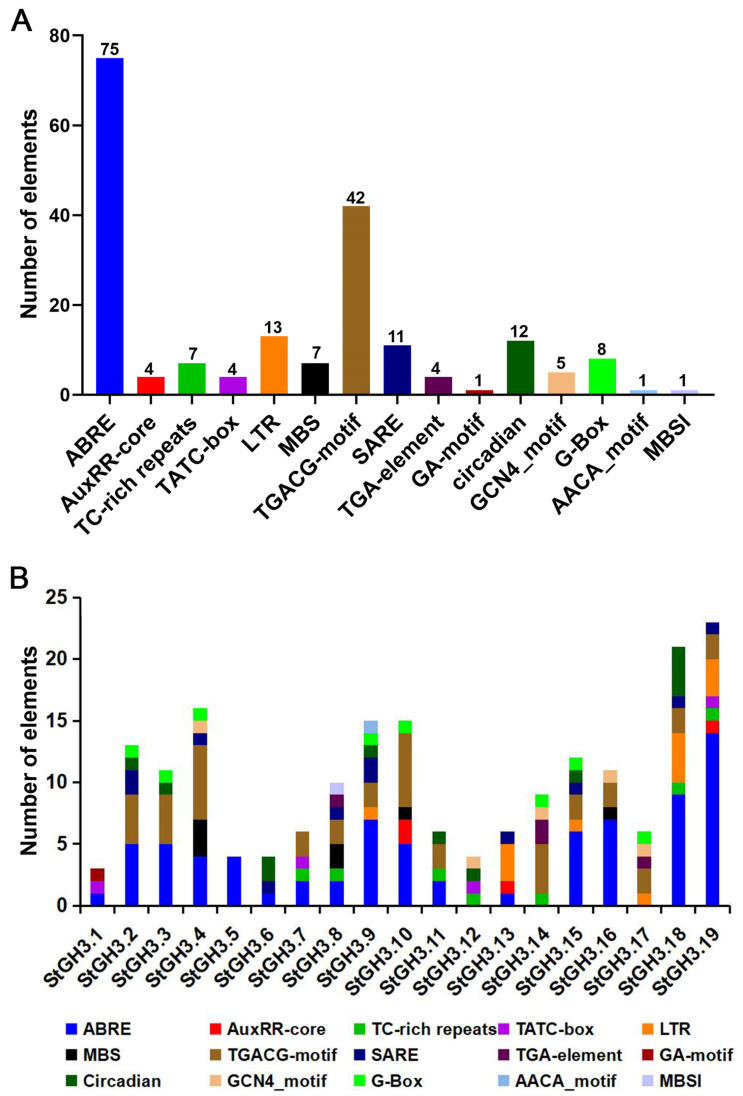
Analysis of 19 cis-acting elements in the *StGH3* promoter region. (**A**) Statistics on the number of stress-related cis-acting elements in all *StGH3* gene promoters. (**B**) Statistics of stress-related cis-acting elements contained in the promoter of each *StGH3* gene.

Cis-acting elements play a crucial role in gene transcription and expression, regulating plant adaptability to various environments through various functions [36]. To further reveal the characteristics of *StGH3s* and predict the possible regulatory pathways involved, the types and numbers of elements in *StGH3* promoter sequences were analyzed (Figure S5). We mainly focused on the environmental response elements. Overall, the *StGH3* gene promoters mainly include hormone response elements (ABRE, TGACG motif, SARE, etc.), stress response elements (MBS, LTR, TC-rich repeats, etc.), and metabolism-related response elements (MBSI and AACA motifs). Among them, the number of ABA response elements (ABREs) was the highest (75), followed by MeJA response elements (TGACG motifs, 42), low-temperature response elements (LTRs, 12), SA response elements (SAREs, 11), and drought-inducible elements (MBS, 7); the least common elements were endosperm-specific elements (AACA motif, 1) and flavonoid biosynthesis elements (MBSI, 1). The hormone response elements are the most widely distributed among the promoters of *StGH3s*; ABA and MeJA response elements are present in almost all promoters (Figure 5). The promoters of *StGH3.8*, *StGH3.9*, and *StGH3.19* contain the most types of elements (7), and *StGH3.5* only contained ABREs. The above results suggested that *StGH3s* may function through certain hormone response pathways.

### 2.7. The Response of StGH3 Genes to Water-Deficit Stress

**Figure 6:**
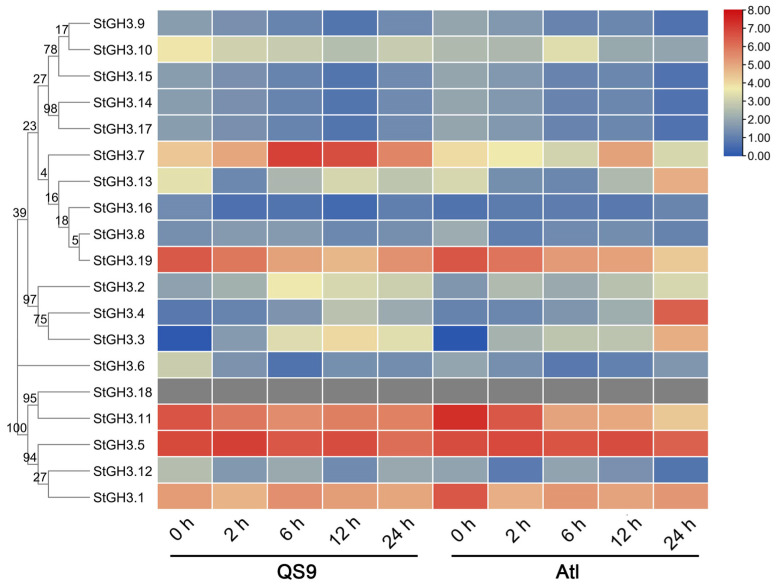
Heatmap showing expression of *StGH3* genes based on RNA-seq data. Heatmap was generated based on log_2_ FPKM. “QS9” and “Atl” represent the drought-tolerant potato variety “Qingshu 9” and drought-sensitive potato variety “Atlantic”, respectively. Three-week-old potato seedlings were treated in 1/2 MS medium with a final concentration of 200 mM mannitol, and the transcriptome was sequenced after 0, 2, 6, 12, and 24 h of stress.

Based on the analysis of *StGH3* gene promoters, we speculated that *StGH3* genes may respond to multiple hormonal and abiotic stresses. Because our team has been devoted to the exploration and regulatory mechanism of water-deficit resistance genes in potato for a long time, we first analyzed the response of *StGH3* genes to water-deficit stress. The results of the transcriptome analysis indicated that there were significant differences in the response of *StGH3* genes to water deficit, which could be divided into three categories according to the response trend (Figure 6). The local expression levels of *StGH3* in the first category were very low, and there are certain changes but no trends with the extension of water deficit; these genes included *StGH3.4*, *StGH3.8*, *StGH3.9*, *StGH3.12, StGH3.14*, *StGH3.15*, *StGH3.16*, *StGH3.17*, and *StGH3.18*. The expression levels of *StGH3* in the second category were relatively high before stress, but the response to water deficit was not obvious; these genes included *StGH3.1*, *StGH3.5*, *StGH3.10*, and *StGH3.13*. For example, the expression level of *StGH3.5* was 47.35-fold higher than that of *StGH3.14*, but the maximum change in *StGH3.5* expression during water-deficit stress was only 1.63-fold. The third category of genes responded significantly to water-deficit stress, including *StGH3.2*, *StGH3.3*, *StGH3.6*, *StGH3.7*, *StGH3.11*, and *StGH3.19*. Although the expression levels of these genes were not the highest before treatment, the changes were relatively significant under water-deficit stress. For example, *StGH3.3* had the lowest expression level at 0 h, but the maximum increase in the two potato materials after water-deficit stress reached 370- and 2374-fold, respectively. At the same time, we also found that the *StGH3* genes in Group III responded more significantly to drought stress, while only *StGH3.7* and *StGH3.19* in Group I showed significant responses to drought stress. The above results indicate that there is a significant difference in the response trend of the *StGH3* genes to drought stress, which also suggests that the drought resistance function of the *StGH3* genes may be different. 

**Figure 7:**
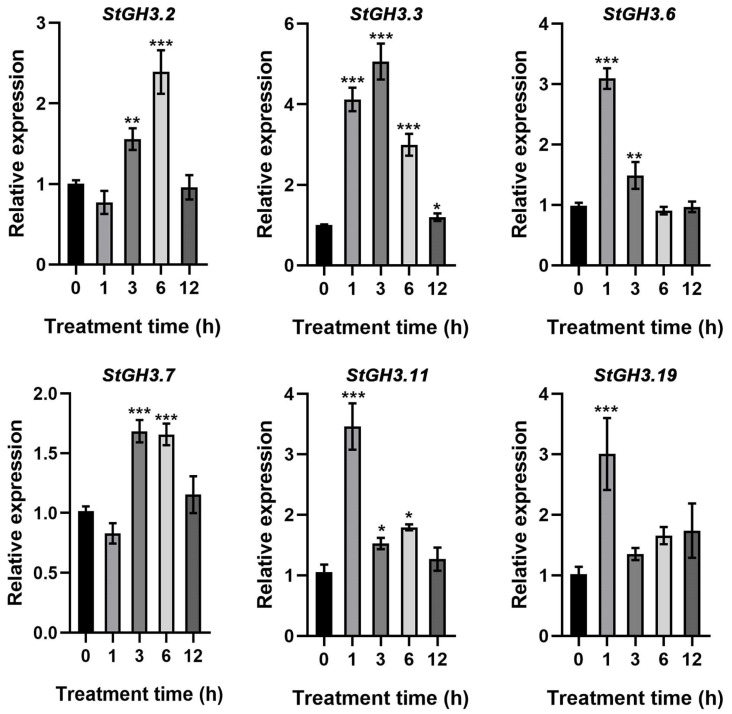
The relative expression level of *StGH3* genes under 200 mM mannitol stress treatments. The 0 h timepoint was taken as a reference to determine relative mRNA levels under stress conditions. Data represent the mean ± SD of three replicates. Data points marked with an asterisk (* *p* ≤ 0.05, ** *p* ≤ 0.01, and *** *p* ≤ 0.001) indicate statistically significant differences between control and stress treatments.

We further verified six *StGH3* genes that significantly responded to water-deficit stress by PCR (Figure 7). The results indicated that the expression levels of these genes were significantly up-regulated under water-deficit stress, but the trends were different. *StGH3.2* and *StGH3.7* showed a similar trend, which increased significantly after 3 h of stress, reaching their maximum after 6 h and returning to the pre-treatment level after 12 h. *StGH3.6*, *StGH3.11*, and *StGH3.19* were significantly up-regulated after 1 h of stress, reaching more than three times that of the 0 h levels. *StGH3.19* quickly returned to the control level after 3 h, while *StGH3.6* and *StGH3.11* decreased significantly after 3 h and 6 h of stress, respectively. It is worth noting that *StGH3.3* was significantly higher throughout the duration of the water-deficit stress. After 1 h of stress, *StGH3.3* expression quickly increased by 4.1 times and reached a maximum value of 4.7 times after 3 h. Although it decreased slightly afterward, it was still significantly higher than the pre-treatment level.

### 2.8. Expression Patterns of Water-Deficit-Stress-Related StGH3 Genes in Different Potato Tissues

**Figure 8:**
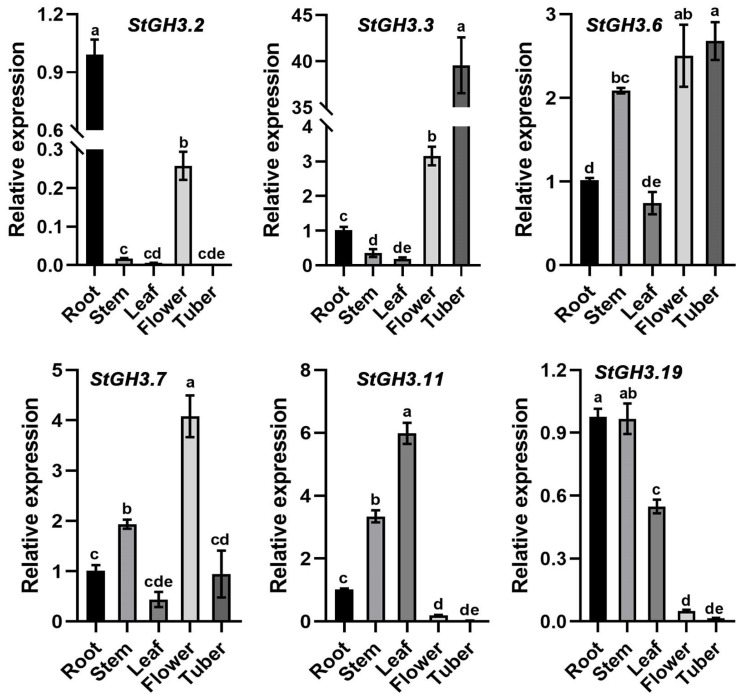
Expression analysis of *StGH3s* in different tissues of flowering potato. The expression level of all genes in roots was set to 1. Error bars are the standard deviation of three measurements. The letters at the top of the bars indicate significant differences between different tissues (α = 0.05, Duncan).

To further investigate the physiological functions of *StGH3* genes, the expression patterns of six water-deficit-stress-related *StGH3* genes in multiple tissues of potato were analyzed (Figure 8). *StGH3.2* was not detected in tubers, while the other five genes were expressed in all tissues. The expression of *StGH3.2* in roots was significantly higher than that in other tissues. The *StGH3.3* expression in tubers was significantly higher than that in other tissues, reaching 37.4-, 110.4-, 203.6-, and 13.1-fold of that in roots, stems, leaves, and flowers, respectively. The expression patterns of *StGH3.6* and *StGH3.7* were slightly different from those of other genes, with smaller differences between tissues; the maximum differences were 3.6- and 9.1-fold, respectively. *StGH3.11* and *StGH3.19* were mainly expressed in roots, stems, and leaves but were extremely low in flowers and tubers. However, some *StGH3* genes in the same sub-group did not show consistent expression patterns, indicating that the functions of these genes may have changed during the evolution of potato. In general, understanding the expression patterns of *StGH3* genes in different tissues can lay a foundation for identifying functional genes in potato.

### 2.9. Effect of Overexpression of StGH3.3 on Water-Deficit Tolerance of Transgenic Tobacco

**Figure 9:**
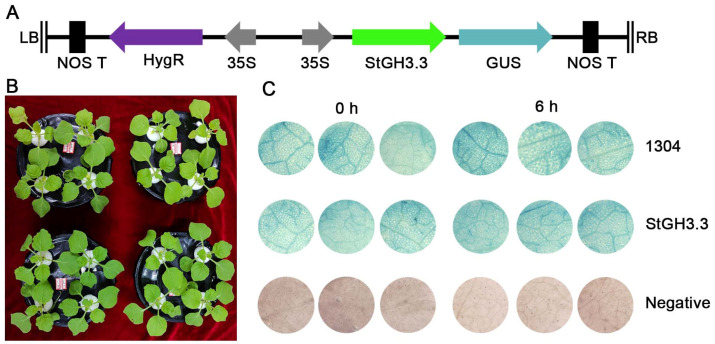
Positive identification of *StGH3.3* transgenic plants. (**A**) Structure of 1304 vector used for genetic transformation. (**B**) Tobacco used for genetic transformation. (**C**) GUS staining of each strain before and after water deficit treatment.

**Figure 10:**
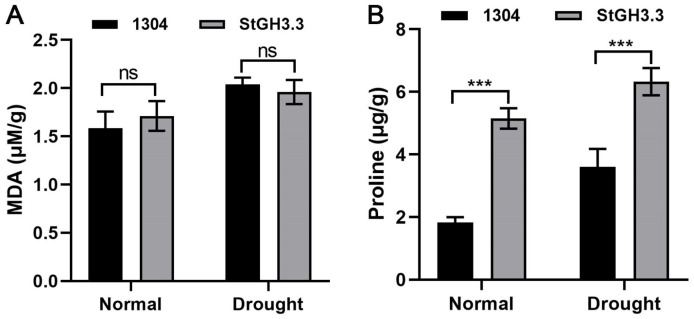
Effect of transient transformation of *StGH3.3* gene on physiological indexes of transgenic plants. (**A**) Accumulation of MDA under normal and water deficit conditions. (**B**) Accumulation of proline under normal and water deficit conditions. The 1304 label refers to transgenic plants overexpressing empty vectors. *StGH3.3* refers to transgenic plants overexpressing the target gene. Data points marked with an asterisk (*** *p* ≤ 0.001) indicate statistically significant differences between control and stress treatments. ns—not significant.

**Figure 11:**
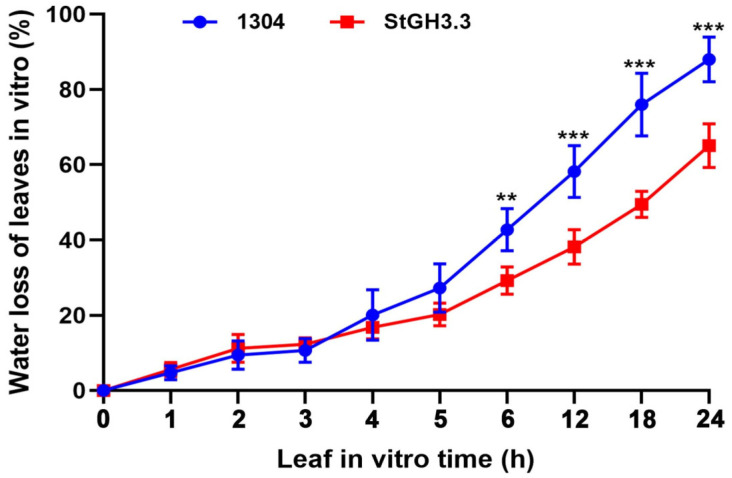
Water loss in leaves of transgenic plants in vitro. After 2 days of transient transformation, leaves of transgenic plants expressing empty vector 1304 and *StGH3.3* were taken and left standing at room temperature. The leaf mass was weighed every hour, and the leaf water loss of each strain was calculated. Data points marked with an asterisk (** *p* ≤ 0.01, *** *p* ≤ 0.001) indicate statistically significant differences between control and stress treatments.

Based on the response of *StGH3* genes to water-deficit stress and tissue-specific expression, we selected *StGH3.3* to study its water-deficit resistance function. Thus, 20-day-old tobacco was tested under the same growth conditions. Two days after transient transformation, we first identified whether the *StGH3.3* was transformed successfully by GUS histochemical staining (Figure 9). We found that dark blue staining could be detected in transient leaves before and after water-deficit stress, while the untransformed leaves were yellowish or white. This result indicated that *StGH3.3* had been successfully expressed in tobacco leaves. Then, the determination of water-deficit resistance physiological indicators indicated that the content of proline in the experimental group was significantly higher than that in the control group under normal and water deficit conditions, while the content of MDA was not significantly different (Figure 10). In addition, we found that the leaves of *StGH3.3* transgenic plants had a slower water loss rate than the control at room temperature. After standing for 6 h, the water loss of control leaves was significantly higher than that of *StGH3.3* transgenic plants at the same time point (Figure 11). Our results suggested that *StGH3.3* may enhance the tolerance of transgenic plants to water-deficit stress by increasing the accumulation of proline.

### 2.10. Expression of Water-Deficit-Stress-Related StGH3 Genes under Multiple Abiotic Stresses

**Figure 12:**
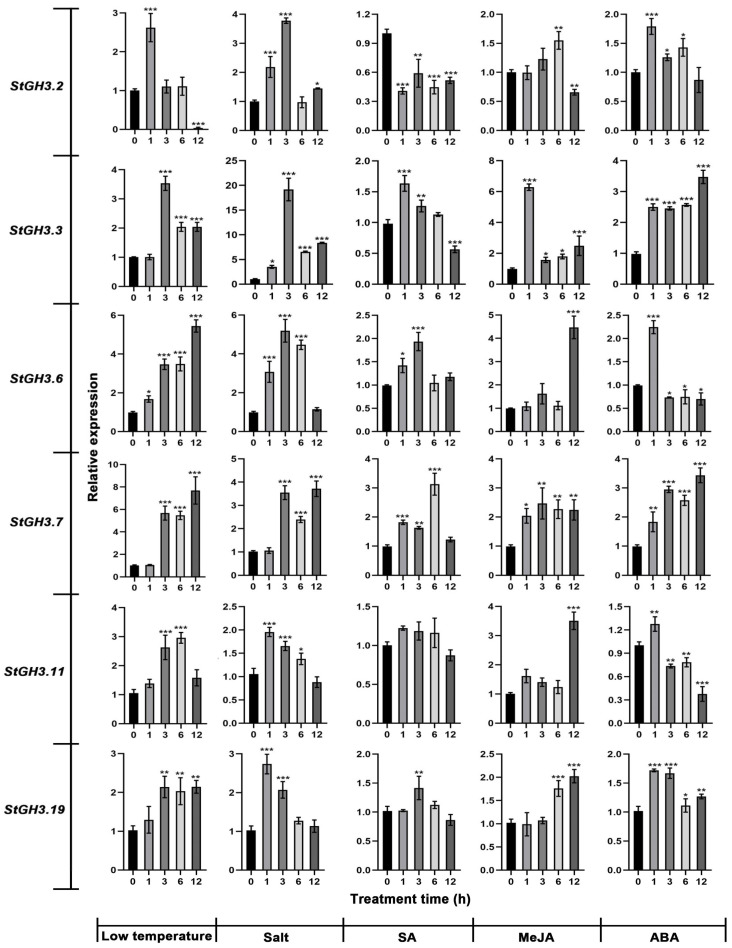
The relative expression level of *StGH3* genes under low temperature and high salt, SA, MeJA, and ABA treatments. The expression level of all genes at 0 h was set to 1. Data represent the mean ± SD of three replicates. Data points marked with an asterisk (* *p* ≤ 0.05, ** *p* ≤ 0.01, and *** *p* ≤ 0.001) indicate statistically significant differences between control and stress treatments.

The analysis of cis-acting elements showed that most *proStGH3s* contained abiotic stress and hormone response elements. Therefore, we further analyzed the response of the six *StGH3* genes to other abiotic stress conditions, including low temperature and high salt, SA, MeJA, and ABA levels (Figure 12). *StGH3.2* and *StGH3.6* responded quickly to low temperatures, and both were significantly up-regulated at 1 h. The expression level of *StGH3.2* reached its maximum at 1 h, while *StGH3.6* continued to increase with the extension of stress and reached its maximum at 12 h. The other *StGH3* genes showed a similar expression trend in response to low temperature: they were significantly up-regulated at 3 h and were maintained at relatively high levels. Under salt treatment, *StGH3.3* responded most violently; it was up-regulated significantly at 1 h and reached its maximum at 3 h, which was 19.8-fold that of the control. The other *StGH3* genes also responded to salt stress; almost all of them exhibit an expression pattern of an initial increase and then a decrease. Under SA treatment, *StGH3* genes exhibited three response modes. *StGH3.2* was significantly reduced in the early stages of stress and remained at a lower level thereafter. *StGH3.3*, *StGH3.6*, and *StGH3.7* all showed a trend of increasing at first and then decreasing. However, *StGH3.11* and *StGH3.19* hardly responded to SA treatment. Under MeJA treatment, all *StGH3* genes except for *StGH3.3* and *StGH3.7* showed a delayed response and were significantly up-regulated after 6 or 12 h. It is worth noting that all *StGH3* genes responded quickly to ABA stress and were significantly up-regulated after 1 h of stress, and *StGH3.2*, *StGH3.6*, *StGH3.11*, and *StGH3.19* reached their maximum levels at 1 h. The above results indicate that the *StGH3* genes not only significantly responds to drought stress, but also to other stresses such as low temperature and high salt, suggesting that the function of the *StGH3* genes may have diversity.

### 2.11. Protein Interaction Network Analysis and GO Analysis of StGH3 Proteins

To further understand the interaction network of StGH3s, we identified proteins that could potentially interact with StGH3s. The results showed that nine StGH3s were predicted to have potential interacting proteins, and 29 proteins were classified into four different interaction networks (Figure S3). Among them, StGH3.5 and StGH3.12 had the most interacting proteins, with 11 and 10, respectively. StGH3.11 had the fewest interacting proteins, with only one. The 20 predicted proteins are also complex and diverse, such as calcium-dependent protein kinase (PGSC0003DMT400028829), salicylic acid-binding protein (PGSC0003DMT400019806), auxin-responsive protein (IAA3), and cytochrome P450 (PGSC0003DMT400078178), etc. In addition, we found that there was a high probability of interaction between StGH3.5 and StGH3.1, so it is speculated that the functions of these two proteins may have a certain dependence.

Meanwhile, GO analyses of the proteins in the network were also performed (Figure S4). The results showed that StGH3s might be involved in multiple complex biological processes, such as response to a biotic stimulus (GO:0009607), regulation of the reactive oxygen species metabolic process (GO:2000377), regulation of stomatal movement (GO:0010119), regulation of growth (GO:0040008), and defense response to bacterium (GO:0042742). Among them, the number of proteins responding to the stimulus process (GO:0050896) was the largest, with 13 proteins. In addition, there are three possible molecular functions, including acid–amino acid ligase activity (GO:0016881), adenylyltransferase activity (GO:0070566), and catalytic activity (GO:0003824). Notably, multiple StGH3s are involved in different biological processes and fulfill diverse catalytic functions. Furthermore, KEGG analysis showed that 16 proteins are involved in the plant hormone signal transduction pathway (sot04075). The results of this study are consistent with existing reports, indicating that plant response to abiotic stress is a complex regulatory network involving multiple metabolic pathways such as signaling and energy.

## 3. Discussion

Environmental stresses, including high salinity, drought, and heavy metal toxicity, cause changes in gene expression and enzyme activities [37]. In response to diverse stress signals, plants alter their growth and development through the regulation of auxin signaling pathways [38]. Studies have shown that auxin is not only related to light-induced morphological composition, apical dominance, and root development but also forms a complex signal transduction network with other hormones [38,39]. GH3 (Gretchen Hagen 3) genes are a family of auxin-driven early response genes that encode a group of proteins that directly affect the homeostasis of other plant hormones [16]. However, the specific function of most *GH3* genes remains unknown. The prediction of all *GH3* genes in the genome became possible with the completion of whole-genome sequencing. At present, the studies on *GH3* genes mainly focused on functional studies of single genes in Arabidopsis, rice, grape, etc. [40]. With the improvement in potato whole-genome sequencing data, the complete sequences of different types of genes can be easily retrieved from the genome. Systematic analysis of the molecular characteristics of *StGH3* family genes and their response patterns to various hormones and abiotic stresses will help us to rapidly screen candidate genes that respond to abiotic stress in potato. GH3-mediated auxin homeostasis is an important part of the complex interaction network between auxin signaling and abiotic stress signaling [41]. In our work, we identified a total of 19 *StGH3* genes. The StGH3 protein sequences have high similarity to GH3 family genes in Arabidopsis and rice [42]. The data suggested that different GH3 proteins might function in the same manner under different microenvironments. The number of *StGH3* family genes is the same as that of Arabidopsis (19 members) and is much more than rice (13 members) [5]. The relatively high amino acid identification of StGH3 suggested that these StGH3 genes may originate from one ancestral sequence. In Arabidopsis, GH3 proteins have been classified into three groups on the basis of sequence similarity and specificity for plant hormones [42]. We also analyzed the phylogenetic relationship among GH3 proteins identified in potato and classified them into two groups (Figure 1). It is worth noting that StGH3 proteins lack group II proteins compared to GH3 proteins in Arabidopsis and rice. This observation is consistent with previous reports of tomato *GH3* belonging to Solanaceae and suggested that group II GH3 proteins might have been lost in potato during the course of evolution [11], which also implied that there may be large variations in the sequence and function of *GH3* among different species. Therefore, the excavation and in-depth study of *GH3* genes in potato will help us to comprehensively analyze the diversity of *GH3* gene functions in plants, which will also lay a very important theoretical foundation for current molecular breeding work.

The expression or transcription of genes is initiated through the upstream regulatory promoter region, which can be considered as a combination of many cis-acting regulatory elements fused with the minimal basic starting unit. The various combinations of regulatory elements endow promoters with characteristics of strength, spatiotemporal specificity, and response to stimuli. Therefore, analyzing the regulatory elements of a target gene promoter can help us predict its response to various stimuli and provide better insights into the functions of the downstream gene. The analysis of *StGH3* gene promoters showed that there were many regulatory elements related to phytohormones, stress, and development (Figure 5). Due to the induction or inhibition of several of these genes by plant hormone signals and/or environmental stress, these data suggest that the expression of these genes may be controlled by different signaling pathways. In addition, these results support previous research that these cis-acting elements may be involved in the regulation of *GH3* genes in this response [37]. AuxRE and TGA elements are two regulatory elements involved in the auxin response. Previous studies have shown that the presence and absence of AuxREs may affect the inducibility of most *GH3* genes [43,44]. Most *GH3* gene promoters contain one or more AuxRE, indicating that they may be responsive to auxin [45,46]. However, only 3 of the 19 *StGH3* genes contain AuxREs, and there are no TGA elements, suggesting that the response of *StGH3* genes to auxin may be different from *GH3* genes in other species. In addition to maintaining the dynamic balance of auxin, *GH3* genes are also widely involved in disease resistance and biotic and abiotic stress responses, which is consistent with the abundance of cis-elements related to stress in their promoters [47,48]. *StGH3* promoters contain many stress-related regulatory elements, including SA-responsive TCA elements and SAREs, ABA-responsive elements (ABREs), MeJA-responsive elements (TGACG motif), defense- and stress-responsive elements (LTR and MBS), and flavonoid biosynthesis elements (MBSI). Salicylic acid is a primary growth hormone that mediates plant disease resistance and abiotic stress responses [24]. Most established *GH3* genes are more or less responsive to SA. However, the *StGH3* gene promoters did not contain a large number of SA-responsive elements; on the contrary, they contained a lot of ABA-responsive elements. Among them, 16 of the 19 *StGH3* gene promoters contain at least one ABA response element. We all know that ABA is the main hormone mediating plant abiotic stress responses [49,50]. Most identified *GH3* genes are more or less responsive to ABA. Therefore, compared with many identified *GH3* genes that mainly respond to SA and MeJA [51], potato *GH3* may be more inclined to be induced by ABA and participate in plant responses to abiotic stress or its own growth through the ABA signaling pathway. In addition, *StGH3* may also play a role in regulating the balance of auxin and ABA, similar to *OsGH3-2* enhancing rice drought and cold resistance by regulating auxin homeostasis and endogenous ABA levels [24]. Further investigation of ABA and auxin crosstalk at the levels of biosynthesis and signaling, as well as their roles in plant growth and responses to abiotic stresses, will help to elucidate the integrative effect of auxin and ABA homeostasis on plant growth and responses to stresses.

Besides auxin, published studies have shown that ethylene, GA, MeJA, and SA also affect the expression levels of several *GH3* genes in various plant species [20,52]. Moreover, *GH3* genes have also been implicated in various biotic and abiotic stress responses, indicating crosstalk between stress and hormone signaling pathways [41]. The physiological effects of various phytohormones are known to be manifested, in part, by alterations to expression levels of genes that are responsive to these hormones [53]. In order to study whether *StGH3* genes are also involved in crosstalk with other phytohormone signals as well as with various environmental stresses, their expression in response to various external stimuli was analyzed (Figure 6, Figure 7 and Figure 11). Here, ABA treatment sharply up-regulated *StGH3.2*, *StGH3.3*, *StGH3.7*, and *StGH3.19*, while slightly repressing *StGH3.6* and *StGH3.11*. Interestingly, the expression patterns of *StGH3* genes under SA treatment were similar to those of the ABA treatment. SA and ABA are known to play key roles in plant defenses, and SA- and JA-dependent defense pathways exhibit crosstalk with each other [54], which suggests that *StGH3* might participate in the crosstalk between these pathways. In rice, wild-type seedlings subjected to various abiotic stresses showed a dramatic increase in the transcription of *OsGH3-1*, *-8*, and *-13* compared to control seedlings [25,47]. In Arabidopsis, WES1 (AtGH3/GH3.5) was strongly induced by ABA and SA treatment [55]. In sorghum, *SbGH3-1*, *-2*, *-4*, *-5*, *-12*, and *-13* were markedly induced in leaves upon salt and drought stress treatments [52]. In this study, some *StGH3s* were markedly induced in response to various phytohormones and abiotic stress treatments, particularly *StGH3.3*, *StGH3.6*, and *StGH3.7* (Figure 12). The changes in the expression response of certain hormones may be due to differences in plant species, indicating new functions that adapt to environmental changes during evolution. In brief, all tested hormones seemed to regulate the expression of *GH3* genes in both a positive and negative manner in potato. This study further supports the hypothesis that these genes might mediate crosstalk among various phytohormone signals and responses to environmental stresses in tomato as well [38]. In addition, studies have shown that most *GH3* genes that respond to hormones or stress belong to group II [51]. However, our results showed that groups I and III of StGH3 also exhibited similar expression trends. 

Plant hormone auxin is essential for plant morphogenesis, such as sunny growth, root morphogenesis, vascular tissue differentiation, axillary bud formation, and floral organ development [56]. The expression analysis of *GH3* gene in different tissues of different plant species at different developmental stages shows that these genes play different roles in plants [11]. A large number of studies on the expression pattern of *GH3* genes in different plants have provided rich references for analyzing *GH3* in potato [18,57]. *TaGH3* was found to be expressed mainly in the roots and leaves and in different amounts [7], while *OsGH3.2* [58] was highly expressed in roots. Many tomato *GH3* genes, including *SlGH3.2*, *SlGH3.3*, *SlGH3.4*, *SlGH3.7*, and *SlGH3.9*, showed the lowest expression levels in the leaves [13]. The expression levels of most *MdGH3* genes in the leaves were much lower than those in the roots [12]. Most *ZmGH3* genes are expressed in the stem, indicating possible roles of *ZmGH3* genes in the growth and development of the stem rather than leaves [8]. Like in other species, *StGH3* in potato also showed tissue-specific expression. In this study, the transcription levels of candidate *StGH3s* in five tissues were determined. Similar to other plant species, *StGH3* genes were differentially transcribed. Some *StGH3* genes, including *StGH3.2*, *StGH3.3*, and *StGH3.7*, have low expression levels in leaves (Figure 8). This observation is similar to previous findings that showed that all *ZmGH3s* and most *MtGH3s* are expressed at lower levels in leaves than in other organs [59,60]. In rice, the *OsGH3-1*, *-4*, *-5*, *-8*, and *-11* genes displayed their highest expression levels in flowers [61], and *OsGH3-8* has been reported as the downstream target of rice MADS-box transcription factor (OsMADS1), which is involved in the patterning of the inner whorl of floral organs [62]. The expression of *StGH3.7* was also distinctly higher in flowers, which may be related to the development of flowers. These findings highlight the role of *StGH3* genes in overall plant development, including various stages of reproductive development. In Arabidopsis, the overexpression of most *GH3* genes in group II changed the balance of active auxin in vivo, forming a dwarf phenotype. This gives us an important clue that the aerial part of potato can be controlled by regulating the expression of *StGH3*. Our data showed that *StGH3.6* and *StGH3.19* were mainly expressed in stems, which indicated that they might play a role in potato growth and development. *GH3* not only participates in the regulation of plant growth and development but also plays an important role in plant responses to abiotic stresses. Overexpression of *OsGH3-2* in rice decreases drought tolerance and ABA levels [24], and cotton *GH3.5* enhances drought and salt tolerance [63]. *MdGH3.6*, as the target gene of *MdMYB94*, plays a negative role in water stress tolerance in apples [64]. The opposite roles of *GH3* genes in different species indicate that *GH3* has diverse functions across plant species. In our study, *StGH3.3*-overexpressing plants had higher proline levels, but there were no effects on MDA levels. MDA is an important intermediate in ROS scavenging, and a high level of MDA is toxic to plant cells. Therefore, MDA is also one of the main indicators for evaluating plant stress resistance. Based on the results of this study, we speculate that the mechanism of *StGH3.3* in potato drought resistance may be different from that of other *GH3* genes. In addition, it is worth noting that *StGH3.3* transgenic plants not only have higher proline content under water deficit conditions but also show the same trend under normal conditions (Figure 10). There were no similar results in the existing studies, which indicated that although GH3 protein is conserved in different species, the molecular mechanism of potato GH3 in stress resistance may be different from other species because of species differences. All plants have different ways of oxidizing reactive oxygen species (ROS), including antioxidant enzymes, such as superoxide dismutase (SOD), catalase (CAT), peroxidase (POD), and ascorbate peroxidase (APX), among others [65]. Therefore, the molecular mechanism of StGH3.3 regulating potato drought tolerance needs further experimental verification. The results also provide a solid theoretical reference for further study on the function of potato gh3 gene and molecular breeding of drought-resistant potato..

## 4. Materials and Methods

### 4.1. Identification of StGH3 Genes in Potato

The largest number of *GH3* genes was found in the potato genome downloaded from the potato genome website (http://spuddb.uga.edu/ (accessed on 15 May 2022)) by two BLASTP methods. The candidate genes were searched by BLASTP using a score value of ≥100 and e-value ≤ e^−10^ [66]. Then, the hidden Markov model (HMM) file from the Pfam protein family database (http://pfam.xfam.org/ (accessed on 15 May 2022)) corresponding to the GH3 domain was downloaded. *GH3* genes were retrieved from the potato genomic database by HMMER3.0. The default parameter was used, and the cutoff was set to 0.01. The PFAM and SMART programs were used to determine the existence of the GH3 core sequence, and genes containing the GH3 domain were further verified by HMMER [67,68]. Finally, 19 *GH3* genes were identified in the potato genome, which were used for further analysis. The sequence length, molecular weight, isoelectric point, and subcellular localization of the GH3 proteins were obtained using the tools of the ExPasy website (http://web.expasy.org/protparam (accessed on 25 May 2022)). 

### 4.2. Gene Structure and Conserved Motif Analysis

We used the gene structure display server (GSDS: http://gsds.cbi.pku.edu.cn (accessed on 3 June 2022)) online program to analyze the exon–intron structure of the *StGH3* genes based on the CDS and the corresponding full-length sequence. The conserved motifs were studied in the encoded GH3 proteins to investigate the structural differences between the *StGH3* genes. We used the GH3 domain sequence of the *StGH3* proteins and the default parameters of ClustalW to compare the protein sequences. The MEME online program (http:/meme.nbcr.net/meme/intro.html (accessed on 7 June 2022)) was used to analyze the protein sequences under the following parameters: the optimum motif width was 15~50, and the maximum number of motifs was 10. 

### 4.3. Chromosomal Distribution and Gene Duplication of StGH3 Genes

The physical location information was obtained from the potato genomic database by Circos, and all *StGH3* genes were mapped to the chromosomes of potato. The potato reference genomic sequence and GFF annotation file were self-aligned by TBtools to analyze the collinearity of *StGH3* members through multiple collinear scanning toolkits (MCScanX). At the same time, the genomic sequence and GFF annotation files of Arabidopsis, soybean, tomato, tartary buckwheat, grape, and sunflower were downloaded from EnsemblPlants (http://plants.ensembl.org/index.html (accessed on 9 July 2022)). Then, the syntenic relationships between *StGH3* genes and *GH3* genes from the selected plant species were determined using Dual Synteny Plotter software (https://github.com/CJ-Chen/TBtools (accessed on 9 July 2022)).

### 4.4. Phylogenetic Analysis of StGH3 Genes

The phylogenetic trees comparing potato, Arabidopsis, rice, and tomato were constructed with the ML method. Multiple amino acid sequences of identified bZIP genes were aligned using MUSCLE. The GH3 protein sequences of Arabidopsis, rice, and tomato were downloaded from NCBI (https://www.ncbi.nlm.nih.gov/ (accessed on 15 August 2022)). In MEGA7, the maximum likelihood method and Poisson model were selected, the option in Rates among Site was set to Gamma Distributed (G), the Site Coverage Cutoff parameter was set to 95%, 1000 Bootstrap tests were carried out, and the evolutionary tree file was output in the “Newick” format [66]. Finally, the phylogenetic tree was visualized using the EvolView website (https://evolgenius.info//evolview-v2/#login (accessed on 16 August 2022)).

### 4.5. Sequence Analysis of StGH3 Gene Promoters

The 2000 bp sequences upstream of the start codon of *StGH3* genes were considered as promoters and extracted by TBtools. Then, the sequences were submitted to the PlantCARE website (http://bioinformatics.psb.ugent.be/webtools/plantcare/html/ (accessed on 7 September 2022)) to predict the type and number of cis-acting elements in the promoter sequences. Finally, the cis-acting elements of *StGH3* genes were visualized by TBtools.

### 4.6. Gene Ontology (GO) Analysis and Protein Interaction Analysis

The GO analysis and protein interaction analysis of StGH3 proteins were completed through the String website (https://cn.string-db.org/cgi/input?sessionId=bAltxfL2OkjD&input_page_show_search=on (accessed on 18 January 2023)). The relevant parameters were set as follows: organisms, Solanum tuberosum; network type, full STRING network; meaning of network edges, evidence; active interactions sources, all options were selected; minimum required interaction score, medium confidence (0.400); max number of interactors to show, no more than 20 interactors; network display mode, interactive svg.

### 4.7. Plant Growth and Abiotic Stress Treatment

The “Qingshu 9” potato strain was acquired from the Agricultural College of Gansu Agricultural University. Test-tube seedlings were cultured in an artificial climate room under the conditions of 16 h/8 h light/dark, 25 °C, and 60% relative air humidity and cultivated to 30 days old for stress treatments [69].

Referring to the treatment methods of potato tissue culture seedlings under abiotic stress in the previous study, the response of *StGH3* genes to various abiotic stresses was analyzed by PCR [69,70]. For the hormone stress, potato test-tube seedlings were sprayed with 100 μM ABA, MeJA, or SA [71]. For the drought and salt stress, potato test-tube seedlings were treated with liquid 1/2 MS medium containing 200 mM mannitol or 100 mM NaCl, respectively [71]. For low-temperature stress, potato test-tube seedlings were moved to a chamber with a 16 h photoperiod at 4 °C. For the blank control, the control plants were mock-treated with alcohol (for hormone stress) and normal liquid 1/2 MS medium (for drought, salt, and low-temperature stresses) under normal conditions. After 0, 1, 3, 6, and 12 h of treatment, the seedlings were collected, frozen rapidly in liquid nitrogen, and stored at −80 °C for RNA extraction. 

### 4.8. Transient Expression of StGH3.3 in Tobacco Leaves

In order to further investigate the function of the *StGH3* genes, we selected *StGH3.3* based on the response of GH3 genes to drought stress and cloned the complete coding sequence. The open reading frame (ORF) of *StGH3.3* was amplified and inserted into the vector 35S-1304-GUS at *Bgl*II and *Spe*I enzyme cut sites. The recombinant plasmid 35S-StGH3.3-GUS was transformed into 20-day-old tobacco leaves in liquid Horland culture by the Agrobacterium tumefaciens-mediated method. Under the same conditions, the empty vector 35S-1304-GUS was transformed and served as the negative control.

Two days after transformation, the whole tobacco plants of the experimental group and the control group were transferred to the liquid Hoagland culture medium containing 200 mM mannitol. After 6 h of stress treatment, the transformation efficiency was quickly identified by GUS histochemical staining, and the contents of malondialdehyde (MDA) and proline (Pro) and the water loss rate of leaves were measured [72]. 

### 4.9. Expression Analysis of StGH3 Genes Using qPCR

The expression patterns of *StGH3s* in tissues and under different stress treatments were analyzed by qRT-PCR. The primers for the *StGH3* genes were designed through the Primer 3 online program (http://frodo.wi.mit.edu/ (accessed on 28 January 2023)) (Table S1). The potato *EF1α* gene was used as the internal reference. Standard RT-qPCR using SYBR Premix Ex Taq II (TaKaRa, Tokyo, Japan) was repeated at least three times on a CFX96 Real-Time System (BioRad, Hercules, CA). The reaction system (10 μL) contained the following: 5 μL PrimeSTAR^®^ Max DNA Polymerase, 1 μL primers (10 mM), 3 μL ddH_2_O, and 1 μL cDNA. The reaction conditions were as follows: 95 °C for 3 min, and 45 cycles of 95 °C for 5 s and 60 °C for 30 s. The results were calculated using the 2^–ΔΔCT^ method, and the relative mRNA expression data were obtained [73].

### 4.10. Statistical Analysis

All the data were analyzed by analysis of variance using the Origin Pro 2019b (OriginLab Corporation., Northampton, MA, USA) statistics program, and the means were compared by the least significant difference test (LSD) at significance levels of 0.05 and 0.01.

## 5. Conclusions

This study comprehensively analyzed the gene structure, chromosome localization, sequence homology, and expression patterns of the *GH3* gene family in potato. We identified a total of 19 *StGH3* genes in potato, which will provide basic information for the functional characterization of *StGH3* genes. Furthermore, expression analysis of the *StGH3* genes in abiotic stress conditions implied that *StGH3s* may participate in the response of potato to stress. Finally, transgenic tobacco with overexpression of *StGH3.3* had enhanced tolerance to water-deficit stress through increased proline accumulation and a reduced leaf water loss rate. Therefore, *StGH3.3* might be a potential target for molecular breeding or genetic editing to produce drought-resistant potato strains. In conclusion, these findings provide insights for predicting the functions of *GH3* genes in drought resistance, and the comprehensive analysis of the results was helpful for screening genes for further functional identification and for genetic improvement of the agronomic traits of potato.

## Data Availability

All data are available within the manuscript.

## Supplementary Materials

The supporting information can be downloaded at https://www.mdpi.com/article/10.3390/ijms242015122/s1.
