# Supplementary material for: Comprehensive Analysis of GH3 Gene Family in Potato and Functional Characterization of StGH3.3 under Drought Stress
Source: Int J Mol Sci. 2023 Oct 12;24(20):15122. doi: 10.3390/ijms242015122 (PMC10606756; doi:10.3390/ijms242015122)
Supplement: Supplementary file 1 [file ijms-24-15122-s001.zip › Suplementary figures.pdf]

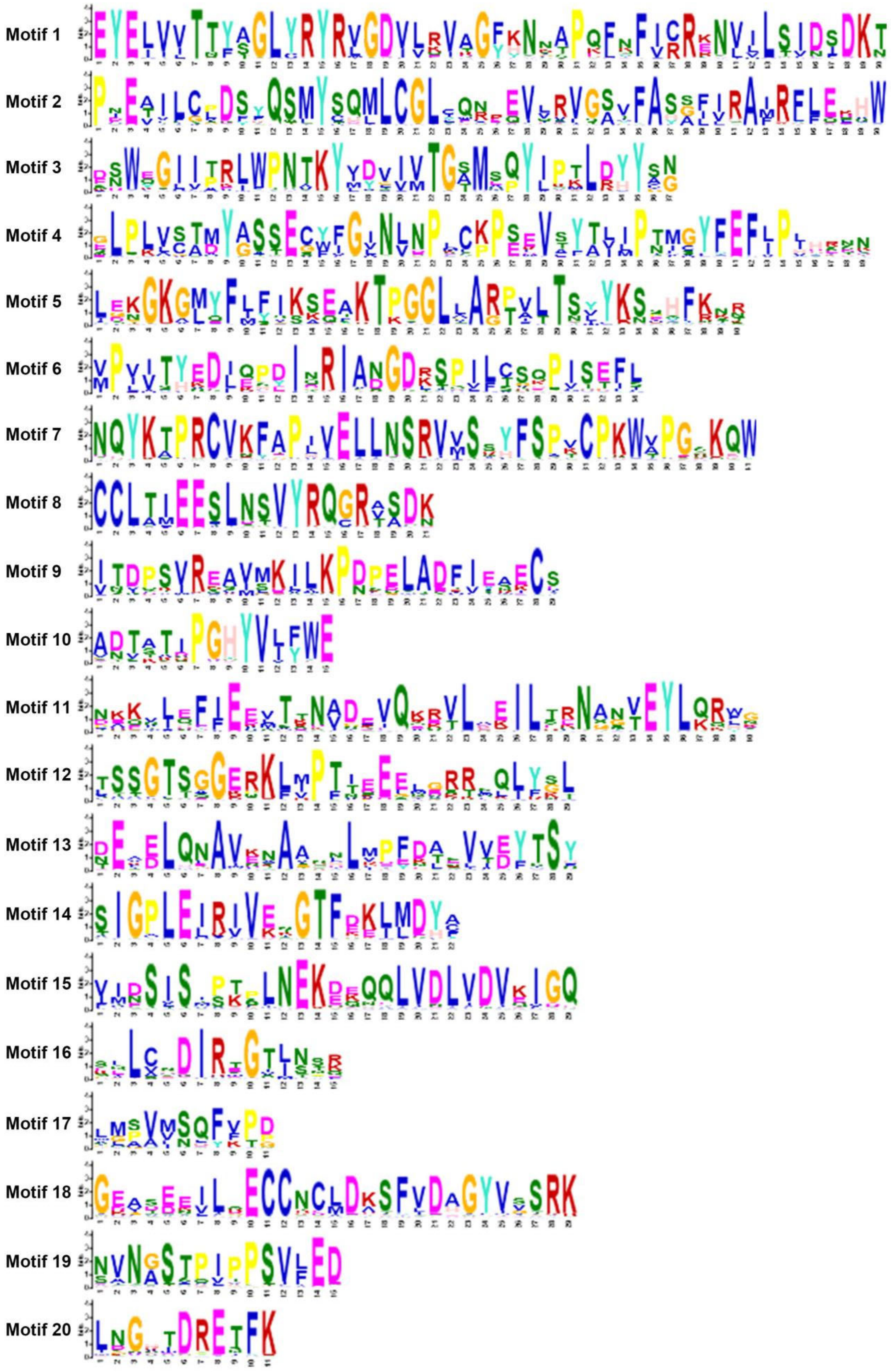

Figure S1. Sequence logo of the GH3 proteins motifs. The height of each amino acid represents the relative frequency of the amino acid at that position

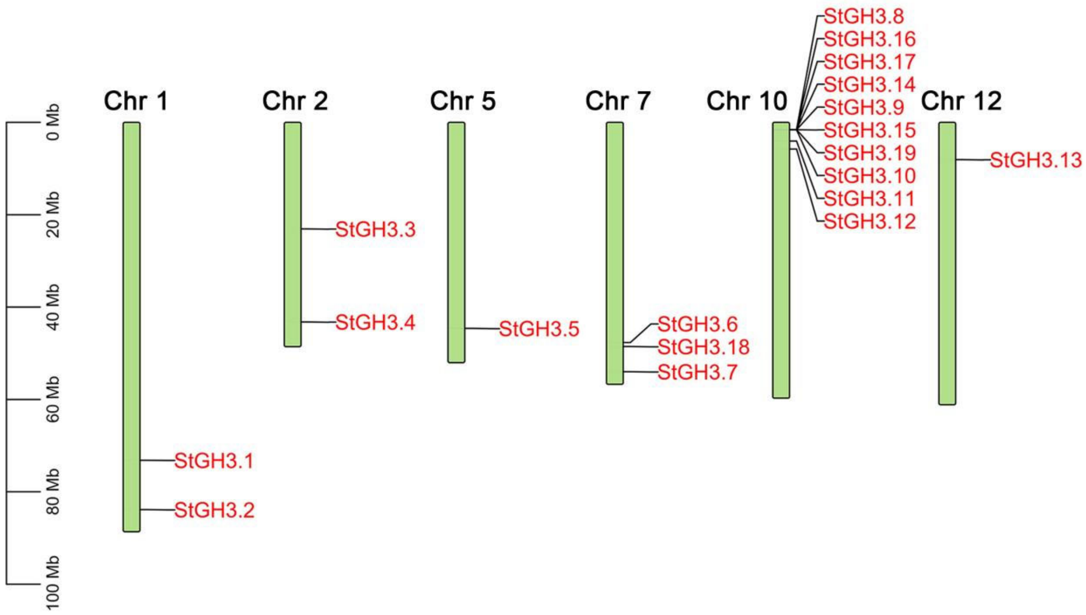

Figure S2. Schematic diagram of chromosome distribution of *StGH3* gene. The chromosome number is displayed on the left side of each chromosome.

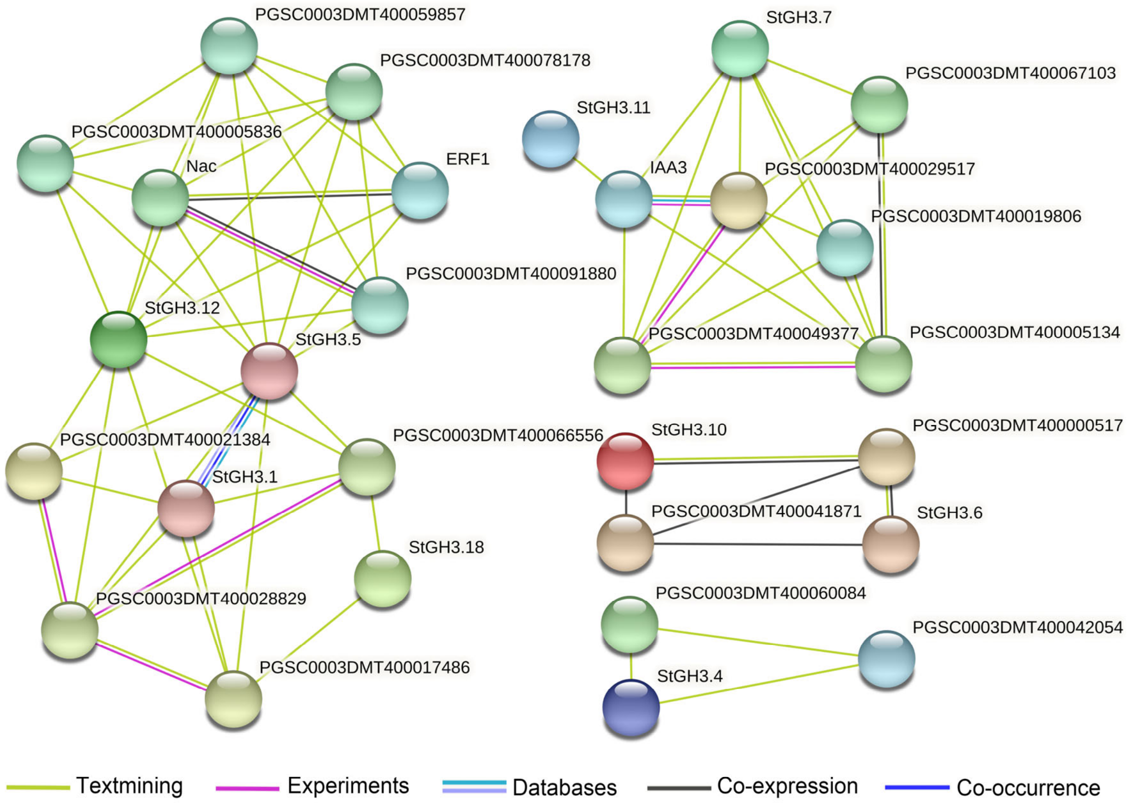

Figure S3. Functional interaction network analysis of StGH3 cascade proteins. Each sphere represents a protein. Different colored lines indicated the way in which the interaction between two proteins connected by lines was verified.

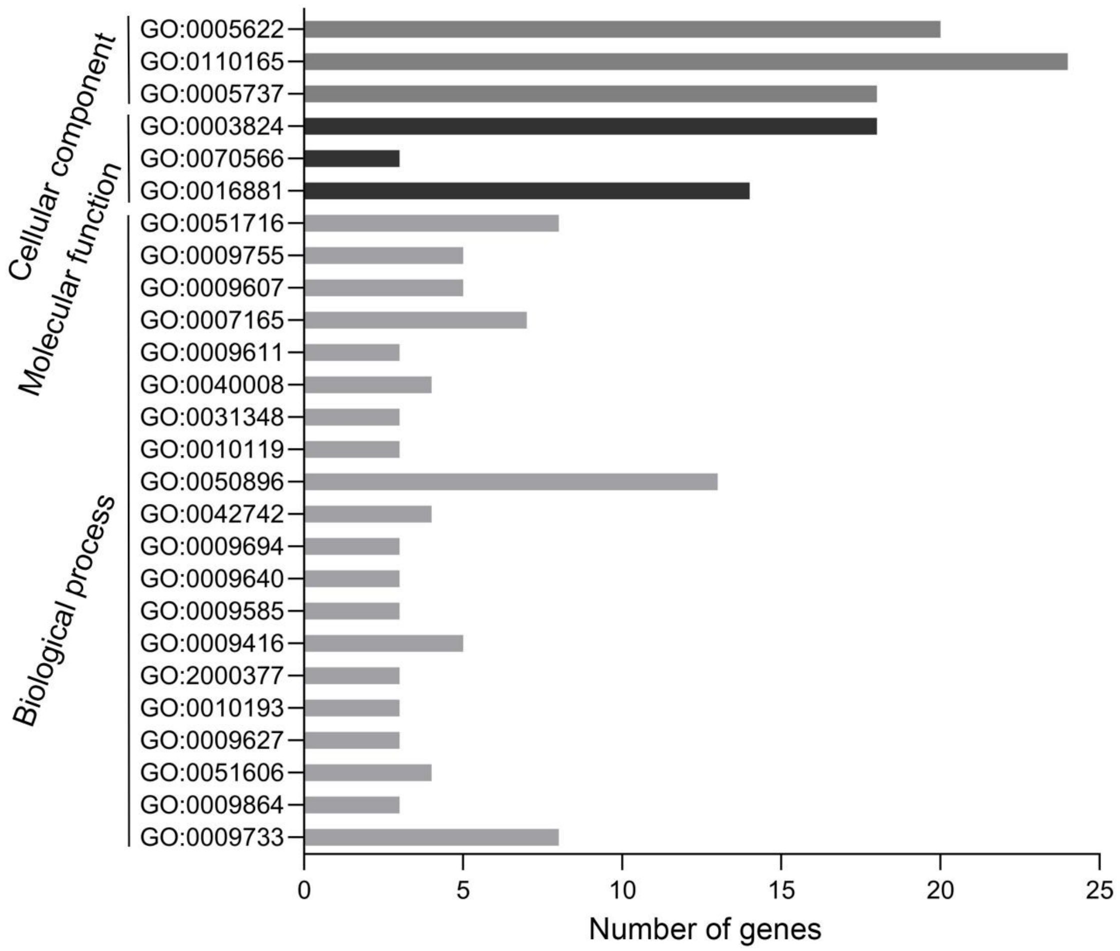

Figure S4. GO analysis of *StGH3s* gene. The horizontal axis represents the number of genes involved in each physiological process, and the vertical axis represents different physiological processes.

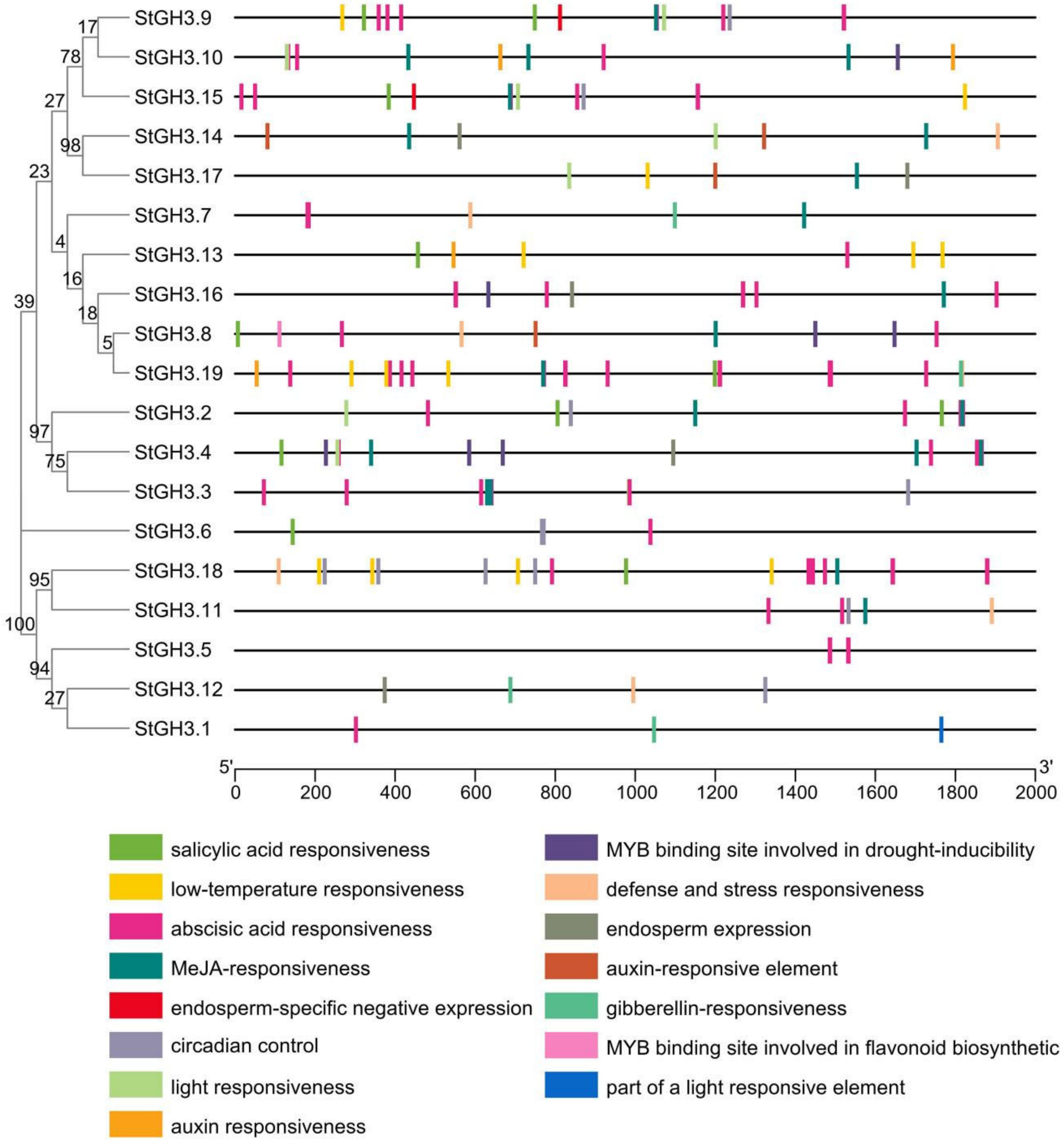

Figure S5. Analysis of 19 *cis*-acting elements in the *StGH3s* promoter region. The same *cis*-elements were displayed in the same color. The black line represents the promoter length of the *StGH3s* gene. The boxes with different colors below represent the functions of each *cis*-acting element.
