# Supplementary material for: Comprehensive Analysis of GH3 Gene Family in Potato and Functional Characterization of StGH3.3 under Drought Stress
Source: Int J Mol Sci. 2023 Oct 12;24(20):15122. doi: 10.3390/ijms242015122 (PMC10606756; doi:10.3390/ijms242015122)
Supplement: Supplementary file 1 [file ijms-24-15122-s001.zip › Suplementary tables.pdf]

Table S1 The sequences of primers were used for *StGH3s* in qRT-PCR

Gene	Sequences
StGH3.2	F: 5'-TGTACGCAGCTTCCGAGTGTTAC-3'
	R: 5'- GGTCAACGAGATTGGTAGGTGAGG-3'
StGH3.3	F: 5'-TGACTCAACCAATCCTCGTGACTC-3'
	R: 5'-GTGAAACCCCGTGACCTGGAG-3′
StGH3.6	F: 5'-TCAGTACAAAGCTCCACGGTGTG-3'
	R: 5'-ACCATTGCTTCTGACCTGGAACC-3'
StGH3.7	F: 5'-CCTCTGCTCTCAACCCATCTCTG-3'
	R: 5'-TCCTCCTCAATCGTTGGCATCAG-3'
StGH3.11	F: 5'- AGTTGTGGAAGGAATTGTGCCATG-3'
	R: 5'- TCTTGATGCCAGCTCTGGATTAGG-3'
StGH3.19	F: 5'-AAGTCGTGCCTGTCATCACCTAC-3'
	R: 5'- TACGGGCTGGGAGCATAGGATAG-3'
Actin	F: 5'- AGGAGCATCCTGTCCTCCTAA-3'
	R: 5'- CACCATCACCAGAGTCCAACA-3'
